# Real-World Anticoagulatory Treatment After Transcatheter Aortic Valve Replacement: A Retrospective, Observational Study on 4,800 Patients

**DOI:** 10.3389/fcvm.2021.780762

**Published:** 2021-12-23

**Authors:** Christopher Hohmann, Marion Ludwig, Jochen Walker, Hendrik Wienemann, Stephan Baldus, Roman Pfister

**Affiliations:** ^1^Department III of Internal Medicine, Faculty of Medicine and University Hospital Cologne, Heart Center, University of Cologne, Cologne, Germany; ^2^InGef–Institute for Applied Health Research Berlin GmbH, Berlin, Germany

**Keywords:** TAVR, anticoagulation, DOAC, antithrombotic therapy, real-world

## Abstract

**Background:** Transcatheter aortic valve replacement (TAVR) has developed to the therapy of choice for patients with symptomatic severe aortic stenosis who are unsuitable for surgical aortic valve replacement and elderly patients with intermediate or high operative risk. However, the optimal anticoagulant therapy post-TAVR still remains a matter of debate.

**Aims:** This study sought to investigate current anticoagulant treatment patterns and clinical outcome in patients undergoing TAVR.

**Methods:** In a retrospective study based on anonymized health claims data of approximately seven million Germans with statutory health insurance (InGef database), anticoagulant treatment regimens were assessed using any drug prescription post discharge within the first 90 days after TAVR procedure. Clinical events between 30 days and 6 months were examined by treatment regime.

**Results:** The study population comprised 4,812 patients with TAVR between 2014 and 2018: 29.4% received antiplatelet monotherapy, 17.8% dual antiplatelet therapy, 17.4% oral anticoagulation (OAC) plus antiplatelet therapy, 12.9% OAC monotherapy, 2.2% triple therapy and 19.2% did not receive any anticoagulatory drugs. Sixty-four percentage of patients with OAC received direct oral anticoagulants (DOAC). Hence, 68% of all patients were treated non-adherent to current guidelines. Forty percentage of patients with OAC prior to TAVR did not have any OAC after TAVR. The adjusted risk of all-cause mortality was significantly increased in patients with OAC (HR 1.40, 95% CI 1.03–1.90, *p* = 0.03) and no anticoagulatory treatment (HR 3.95, 95% CI 2.95–5.27, *p* < 0.0001) when compared to antiplatelet monotherapy.

**Conclusions:** This large real-world data analysis demonstrates substantial deviations from guideline recommendations and treatment after TAVR. Considering relevant differences in clinical outcome across treatment groups, major effort is warranted to examine underlying causes and improve guideline adherence.

## Introduction

Transcatheter aortic valve replacement (TAVR) has developed to the therapy of choice for patients with symptomatic severe aortic stenosis who are unsuitable for surgical aortic valve replacement and elderly patients with intermediate or high operative risk (1–3). A major clinical challenge remains to balance risk of bleeding and cardiovascular events, given a stroke risk after discharge of up to 3%, silent valve thrombosis of up to 20% (4–6) and major bleeding events in up to 13% (7, 8).

The optimal anticoagulant therapy post-TAVR still remains a matter of debate. Current guidelines during our study period between 2014 and 2018 recommended the use of dual antiplatelet therapy (DAPT) for 3 to 6 months in patients without an indication for oral anticoagulation (OAC). However, this recommendation was mainly based on expert consensus (9). Recently, updated guidelines have recommended antiplatelet monotherapy after TAVR according to the results of a recent randomized controlled trial (10). Important of note, elderly patients undergoing TAVR usually exhibit numerous comorbidities which additional affect vascular and thromboembolic risk. For instance, acute coronary syndrome or coronary stent implantation prior to TAVR requiring longer or more intense dual antiplatelet therapy is reportedin nearly one-third of patients.

A similar proportion of patients have atrial fibrillation or other conditions requiring chronic OAC therapy (3, 11). Current practice guidelines recommend a vitamin-K antagonist (VKA) either alone or in combination with aspirin or clopidogrel in this situation (1, 10). Although direct oral anticoagulants (DOAC) are currently not recommended after TAVR and were associated with worse outcomes compared to VKA in patients after mechanical valve replacement, the superior safety profile, the ease of use and the lack of data on harm after TAVR might tempt the use of DOAC in clinical routine (12–14).

Taken together, there is much uncertainty regarding anticoagulant therapy in the individual patient after TAVR. It is unknown but of major relevance how this translates into guideline adherence in the real-world treatment of TAVR patients, considering the major impact of anticoagulant therapy in elderly and multimorbid patients (15). The aim of the present study was to assess current anticoagulant treatment patterns in the context of guideline adherence and clinical outcome in patients undergoing TAVR using longitudinal German Statuary Health Insurance claims data.

## Methods

### Study Design and Data Source

This non-interventional retrospective cohort study was based on anonymized health claims data of approximately seven million Germans with statutory health insurance (InGef database). The InGef database provides longitudinal data on the utilization of services on a case-by-case individual level. In brief, the database includes demographic information, information on outpatient healthcare services and data related to hospital treatment, including admission and discharge dates, diagnoses, operations and interventions (OPS codes) as well as prescription and dispensation of reimbursed medications. All diagnoses in the database were coded according to the International Classification of Diseases (ICD), 10th Revision, German Modification (ICD10-GM). Data on outpatient prescriptions of reimbursed drugs comprise information on the prescription, the date of prescription and the pharmaceutical reference number. The database has a high external validity regarding morbidity, mortality and drug prescriptions (16). All patient identifiers were either fully encrypted or removed from the database which is therefore compliant with the German data protection regulations. As no patient contact was made and patient information was deidentified, Institutional Review Board Approval was not required.

### Study Population

We identified adult patients (≥18 years of age) with available information on age and gender who received TAVR for the first time within the study period from 01 January 2014 to 31 December 2018 using OPS codes 5–35a.00, 5–35a.01, 5–35a.02, 5–35a.03, and 5–35a.04. The date of TAVR was defined as the index date. Patients were required to have a continuous health plan enrollment for 6 months pre-index (baseline period of 180 days used for assessment of baseline characteristics of morbidity and medication pre TAVR) as well as 6 months post-index or death. Patients with a history of mechanical heart valve replacement or previous TAVR and patients with DOAC dosages that are not approved for prophylaxis of thromboembolic events in Germany were excluded.

Depending on prescriptions of any of the following antithrombotic or anticoagulation drugs at any time within the period of 90 days post-TAVR (postprocedural anticoagulation regime), patients were grouped into a total of 6 mutually exclusive regimens: no antithrombotic/anticoagulant therapy, single antiplatelet therapy (aspirin, clopidogrel, prasugrel, or ticagrelor), dual antiplatelet therapy (aspirin in combination with either clopidogrel, prasugrel or ticagrelor), single oral anticoagulation (with a DOAC [apixaban, edoxaban, dabigatran, or rivaroxaban] or VKA [phenprocoumon]) (OAC mono), oral anticoagulation (with a DOAC [apixaban, edoxaban, dabigatran, or rivaroxaban] or VKA [phenprocoumon]) plus aspirin or clopidogrel (OAC duo) and oral anticoagulation (with a DOAC [apixaban, edoxaban, dabigatran, or rivaroxaban] or VKA [phenprocoumon]) plus aspirin and clopidogrel (OAC triple). Patients who received a prescription that could not be categorized into one of the aforementioned regimens were classified as undefinded therapy, for instance patients who were prescribed both phenoprocoumon and a DOAC or more than one distinct DOAC.

### Clinical Endpoints

The primary effectiveness outcomes were (1) the combined endpoint of ischemic stroke and systemic embolism, (2) mechanical complications by artificial heart valve, and (3) major adverse cardiovascular events (MACE) defined as a combined endpoint of cardiovascular mortality, myocardial infarction and ischemic stroke, and (4) death from any cause. The primary safety outcomes were intracranial bleeding, major extracranial bleeding and gastrointestinal bleeding. Intracranial bleeding was defined as subarachnoidal bleeding, intracerebral bleeding and other non-traumatic and traumatic intracranial bleeding. Major extracranial bleeding was defined as a bleeding with anemia, hemothorax, conjunctival hemorrhage, retinal hemorrhage, unspecified, recurrent and persistent haematuria, hemorrhage from respiratory passages, haemarthrosis as well as other abnormal uterine and vaginal bleeding. The outcomes were identified using ICD-10-GM hospital main and secondary discharge diagnosis as well as ambulatory verified diagnoses (Supplementary Table 1). The prementioned endpoints were evaluated for the period of >30 days up to 6 months after TAVR, respectively, since events during the early postprocedural period are usually attributable to the procedure itself and less likely due to the anticoagulation medication which is usually initiated several days after the procedure.

### Statistical Analysis

Data analysis was performed by InGef–Institute for Applied Health Research Berlin GmbH, Germany. The primary outcome was the postprocedural anticoagulation regime. Baseline characteristics of the study population were reported as percentages or mean ± standard deviation. Statistical significance across groups was examined using chi-square test for categorical variables and one-way analysis of variance (ANOVA) for metric variables, respectively. Secondary outcomes were compliance with current guidelines (i.e., DAPT or any VKA containing OAC regime, but no DOAC), postprocedural termination of OAC and postprocedural initiation of OAC without justifying diagnosis. Secondary analysis on the prevalence of postprocedural anticoagulation regimes was performed excluding patients who died within the first 30 days after TAVR. The reason was the lack of data on anticoagulant treatment during the hospitalization and potential drug intake provided by the hospital for the early post-discharge days and in consequence the uncertainty of assignment of such patients to respective anticoagulation regimes, in particular the “no anticoagulation” category.

Unadjusted event rates were calculated by dividing the number of events by the person time and were reported per 100 person-years. Cox proportional-hazard regression models were used to estimate treatment effects of dual antiplatelet therapy, any OAC (total of OAC mono, OAC duo, OAC triple) and no anticoagulation for all-cause mortality in the period >30 days up to 6 months after TAVR using single antiplatelet therapy as the reference group. Models were adjusted for prespecified baseline demographics and clinical factors only for all-cause mortality which had a sufficient number of events. Variables for inclusion in the model were selected based on established evidence on the effect of the specific variable on the choice of treatment and mortality. To estimate the magnitude of underdetection of postprocedural prescription of anticoagulation using a 90 days interval, we examined how many patients received a follow-up prescription of VKA/DOAC or antiplatelet agent, respectively, within an observation period of 120 days after their last preprocedural prescription in the two patient groups with intake of single antiplatelet therapy or VKA/DOAC prior to TAVR and no respective follow-up prescription within the 90 days post TAVR. All statistical analyses were performed using SAS software version 9.4 (SAS Institute GmbH, Heidelberg, Germany).

## Results

### Postprocedural Anticoagulant Treatment

The study population comprised 4,812 patients with TAVR between 2014 and 2018. Mean age was 81 ± 6 years and 55% were male. Mean Charlson Comorbidity Index was 4.4 + 3.0. The most frequent comorbidities were hypertension (91%), coronary heart disease (61%), congestive heart failure (50%), diabetes mellitus (45%) and atrial fibrillation (37%) (Table 1).

**Table 1:** Baseline characteristics of patients after TAVR.

	**Monotherapy (ASS/Clopidogrel)**	**DAPT**	**OAC mono**	**OAC duo**	**OAC triple**	**No anticoagulation**	**Undefined therapy**	**Total study population**	**overall *p*-value**
	***n =* 1,414**	***n =* 854**	***n =* 619**	***n =* 837**	***n =* 104**	***n =* 926**	***n =* 58**	***n =* 4,812**	
Age (mean ± SD)	80.6 (6.4)	80.3 (6.5)	81.9 (5.4)	81.5 (5.8)	80.6 (6.0)	80.7 (6.7)	81.8 (5.0)	80.9 (6.2)	0.00
Male (%)	798 (56.5)	443 (51.8)	335 (54.1)	453 (54.1)	60 (57.7)	534 (57.7)	29 (50.0)	2528 (55.1)	0.20
Charlson Comorbidity Index (mean ± SD)	4.2 (2.9)	4.2 (3.1)	4.5 (2.9)	4.5 (3.0)	4.4 (2.7)	4.6 (3.0)	4.4 (2.5)	4.4 (3.0)	0.11
CHA_2_DS_2_-VASc-Score (mean ± SD)	4.6 (1.3)	4.5 (1.3)	4.8 (1.3)	4.8 (1.3)	4.8 (1.3)	4.7 (1.4)	5.1 (1.4)	4.7 (1.3)	0.00
modified HAS-BLED-Score (mean ± SD)	2.9 (1.0)	2.9 (1.0)	3.0 (1.0)	2.9 (1.0)	2.9 (0.9)	3.0 (1.0)	3.0 (0.9)	2.9 (1.0)	0.25
Renal insufficiency (%)	413 (29.2)	251 (29.4)	203 (32.8)	260 (31.1)	29 (27.9)	336 (36.3)	18 (31.0)	1415 (30.8)	0.01
Dementia (%)	103 (7.3)	62 (7.3)	54 (8.7)	63 (7.6)	9 (8.6)	61 (6.6)	<5	340 (7.4)	0.28
History of ischemic stroke/TIA (%)	90 (6.4)	64 (7.5)	55 (8.9)	63 (7.6)	10 (9.6)	81 (8.7)	7 (12.1)	347 (7.6)	0.21
Myocardial infarction <12 months (%)	99 (7.0)	46 (5.4)	43 (6.9)	63 (7.6)	10 (9.6)	59 (6.4)	5 (8.6)	315 (6.9)	0.49
Coronary heart disease (%)	891 (63.0)	461 (54.0)	383 (61.9)	531 (63.4)	59 (56.7)	558 (63.5)	39 (67.2)	2808 (61.2)	0.00
History of coronary angioplasty (PCI)/Stenting (%)	60 (4.2)	33 (3.9)	8 (1.3)	15 (1.8)	<5	25 (2.7)	<5	139 (3.0)	0.00
Congestive heart failure (%)	635 (44.9)	356 (41.7)	375 (60.6)	451 (53.9)	49 (47.1)	486 (52.5)	33 (56.9)	2260 (49.3)	0.00
Hypertension (%)	1294 (91.5)	746 (87.4)	570 (92.1)	772 (92.7)	98 (94.2)	851 (91.9)	56 (96.5)	4180 (91.1)	0.00
Cancer (%)	374 (26.5)	228 (26.7)	166 (26.8)	191 (22.9)	30 (28.8)	252 (27.2)	9 (15.5)	1200 (26.2)	0.15
Arteriosclerosis (%)	407 (28.8)	217 (25.4)	160 (25.8)	245 (29.3)	31 (29.8)	264 (28.5)	17 (29.3)	1263 (27.5)	0.46
Diabetes mellitus (%)	639 (45.2)	377 (44.1)	285 (46.0)	375 (44.8)	53 (50.1)	425 (45.9)	31 (53.4)	2075 (45.2)	0.72
Obesity (%)	332 (23.5)	185 (21.7)	181 (29.2)	229 (27.5)	30 (28.8)	222 (24.0)	20 (34.5)	1147 (25.0)	0.00
History of any bleeding event (%)	172 (12.2)	102 (11.9)	108 (17.4)	131 (15.7)	12 (11.5)	161 (17.4)	11 (19.0)	662 (14.4)	0.00
Moderate or severe hepatic insufficiency (%)	<5	<5	<5	<5	<5	<5	<5	7 (0.1)	0.32
Atrial fibrillation (%)	282 (20.0)	92 (10.8)	410 (66.2)	530 (63.3)	58 (55.8)	381 (41.1)	32 (55.2)	1687 (36.8)	0.00
Previous venous thromboembolism (%)	42 (3.0)	25 (2.9)	36 (5.8)	48 (5.7)	5 (4.8)	34 (3.7)	<5	184 (4.0)	0.00
ACE inhibitors/angiotensin receptor antagonist (%)	508 (35.9)	297 (34.8)	232 (37.5)	331 (39.6)	42 (40.4)	345 (37.3)	25 (43.1)	1701 (37.1)	0.38
NSAIDs (%)	321 (22.7)	185 (21.7)	127 (20.5)	170 (20.3)	19 (18.3)	175 (18.9)	16 (27.6)	970 (21.1)	0.28
Betablocker (%)	835 (59.1)	443 (51.9)	441 (71.2)	565 (67.5)	71 (68.3)	583 (63.0)	38 (65.5)	2834 (61.8)	0.00
Diuretics (%)	762 (53.9)	411 (48.1)	419 (67.7)	538 (64.6)	67 (64.4)	605 (65.3)	33 (56.9)	2676 (58.3)	0.00
Antipsychotics (%)	37 (2.6)	25 (2.9)	14 (2.3)	26 (3.1)	<5	28 (3.0)	<5	128 (2.8)	0.41
Proton pump inhibitors (%)	583 (41.3)	324 (38.0)	271 (43.8)	342 (40.9)	40 (38.5)	398 (43.0)	22 (37.9)	1872 (40.8)	0.30
Statins (%)	727 (51.4)	407 (47.7)	299 (48.3)	410 (49.0)	43 (41.3)	477 (51.5)	23 (39.7)	2279 (49.7)	0.12
All- cause mortality <30 days after procedure (event rate)	<5 (n.a.)	<5 (n.a.)	<5 (n.a.)	<5 (n.a.)	<5 (n.a.)	219 (70.0)		

*Baseline characteristics were determined in the 180 days prior to the respective TAVR procedure. Data are n (%), unless otherwise indicated. Data are not shown in cells that contain fewer than five patients. ACE, angiotensin-converting enzyme; ASS, acetylsalicylic acid; NSAID, non-steroidal anti-inflammatory drug; SD, standard deviation*.

**Figure 1:**
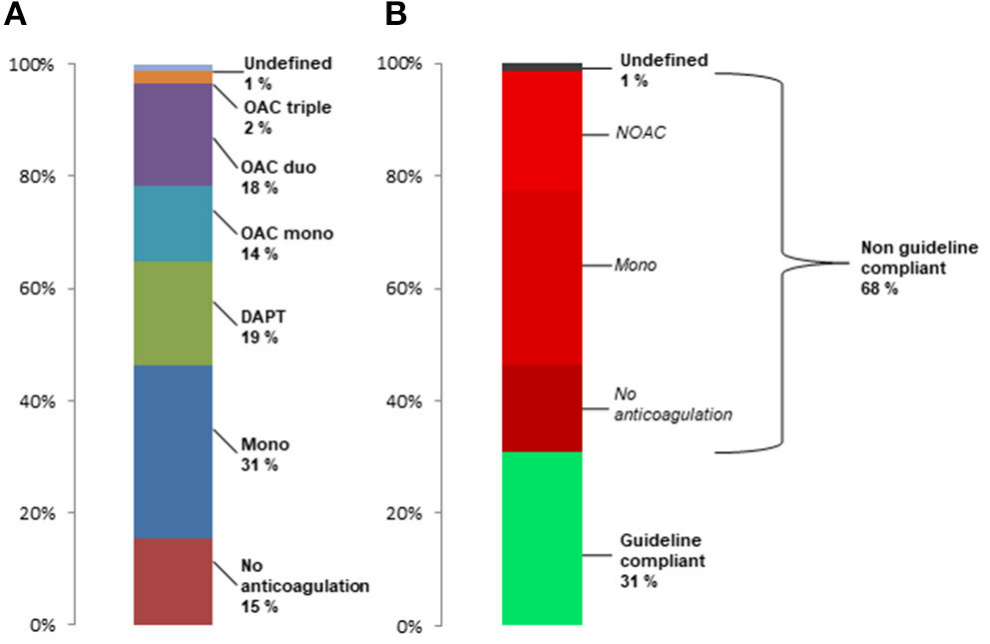
Frequency of post-TAVR anticoagulant treatment regimes in the total population **(A)** and rate of treatments incompliant to current guideline recommendation in patients surviving first 30 days after TAVR **(B)**.

Based on all drug prescriptions during the first 90 days after TAVR the postprocedural anticoagulation regime was assessed for every patient (Figure 1A). The majority of patients received antiplatelet monotherapy (*n* = 1,414, 29.4%), followed by DAPT (*n* = 854, 17.8%), OAC duo (*n* = 837, 17.4%), OAC mono (*n* = 619, 12.9%) and OAC triple (*n* = 104, 2.2%). A total of 926 patients (19.2%) did not receive any prescription of an anticoagulant drug and 58 patients (1.2%) had an undefined anticoagulation regime based on the combination of drug prescriptions.

Baseline characteristics of the total study population and by postprocedural anticoagulation regime are presented in Table 1. The frequency of individual characteristics differed significantly between treatment groups with regard to age, renal insufficiency, coronary heart disease, history of coronary angioplasty, congestive heart failure, arterial hypertension, atrial fibrillation, obesity, history of any bleeding event, previous venous thromboembolism and intake of beta-blocker and diuretics. For example, atrial fibrillation and previous venous thromboembolism were 2- to 3-fold more common in patients with a regime including OAC, and a history of any bleeding event was highest in patients with no anticoagulation and OAC mono. Regarding platelet inhibition, single and dual antiplatelet therapy was associated with a more frequent history of coronary angioplasty.

### Guideline Adherence

According to current guideline recommendations during the study period between 2014 and 2018, 48.6% of patients were formally undertreated with either no anticoagulation or single antiplatelet therapy. When excluding patients who died during the first 30 days after TAVR (*n* = 225), still 46.2% were undertreated with 30.8% having only antiplatelet monotherapy and 15.4% having no prescription for anticoagulant drugs (Figure 1B). Furthermore, 21% of all patients who survived the first 30 days had DOAC after TAVR, corresponding to 64% of patients with postprocedural OAC. In summary, 68% of patients had anticoagulant treatments which are not compliant with current recommendations.

**Figure 2:**
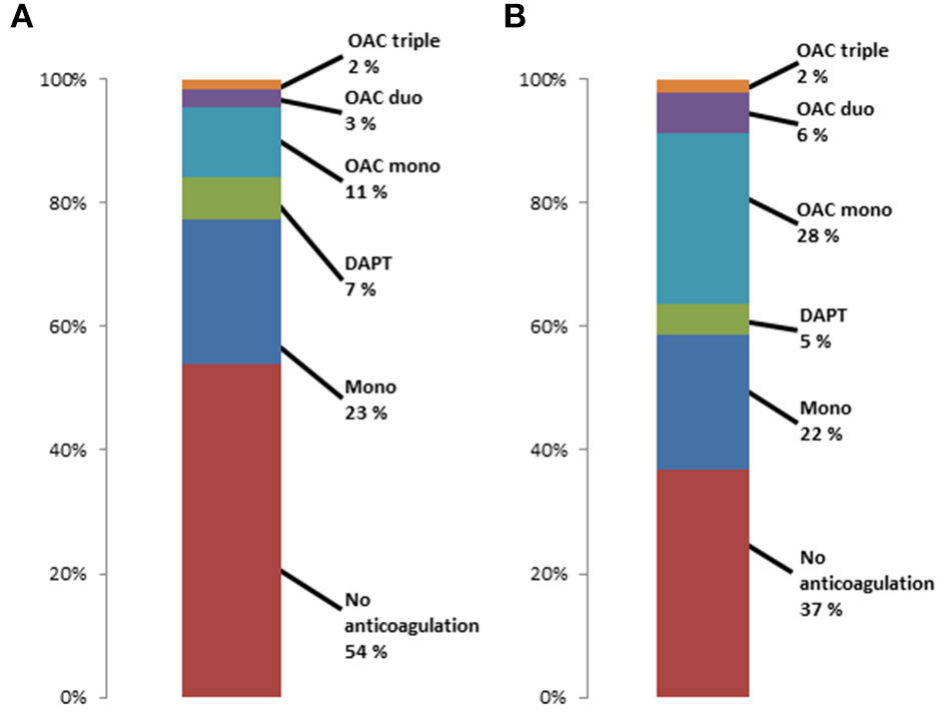
Preprocedural anticoagulant regimes in patients surviving first 30 days after TAVR with **(A)** postprocedural antiplatelet monotherapy and **(B)** postprocedural no antiplatelet or anticoagulation therapy.

Figure 2 shows the distribution of preprocedural anticoagulation regimes for patients who survived the first 30 days after TAVI and had postprocedural antiplatelet monotherapy (A) or no anticoagulation (B). More than 50% of patients with postprocedural antiplatelet monotherapy had no anticoagulant medication prior to TAVR, and 22.7% had a more extensive anticoagulation such as DAPT or OAC. 37% of patients without any postprocedural anticoagulation had no anticoagulant medication prior to TAVR, and about 36% had OAC prior to TAVR.

From the perspective of the recently updated 2021 ESC/EATCS guidelines, 19.2% of patients were formally undertreated with no antithrombotic or anticoagulant therapy. With the additional consideration of patients who were prescribed a DOAC after TAVR, a total of 39% of patients had anticoagulant treatments which were not compliant with updated guideline recommendations.

### Changes in OAC and Type of OAC From Pre- to Post-procedural

**Figure 3:**
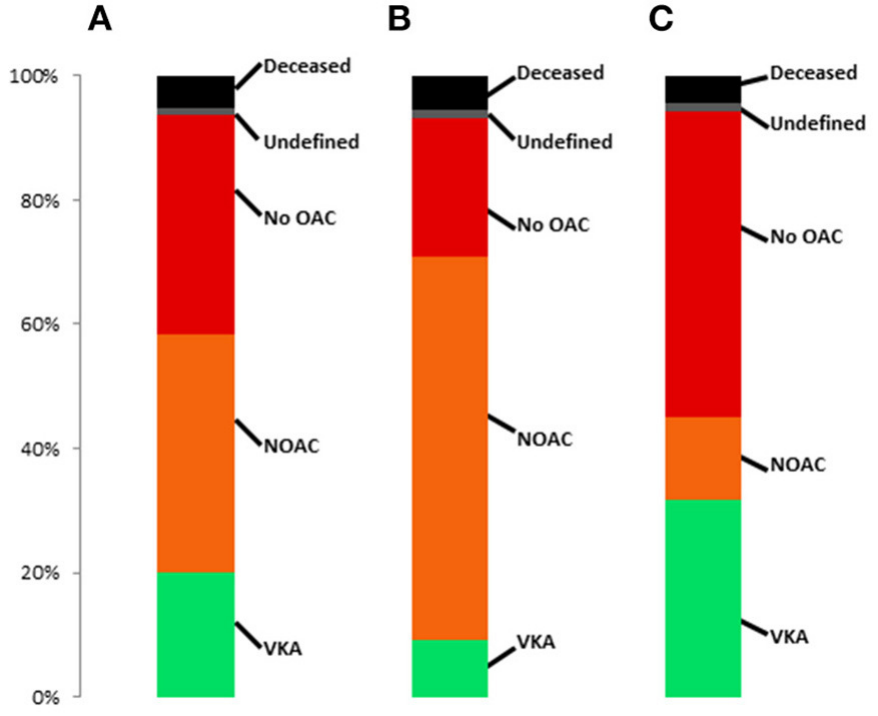
Frequency of post-TAVR anticoagulant treatment regimes in patients with **(A)** OAC prior to TAVR, **(B)** DOAC prior to TAVR and **(C)** VKA prior to TAVR.

1,551 (32.2%) patients had any OAC before TAVR, with similar use of DOAC (*n* = 806, 52.0%) and VKA (*n* = 745, 48.0%). The respective postprocedural type of OAC in patients with any preprocedural OAC, preprocedural NOAC and preprocedural VKA is presented in Figure 3. Almost 40% of patients did not have any OAC prescription post-procedurally. The rate was significantly higher in patients with prior VKA treatment compared to prior DOAC treatment (*p* < 0.001). When excluding patients who died during the first 30 days after TAVR, still 37.0% did not have any postprocedural OAC prescription.

A total of 609 patients were newly prescribed OAC after TAVR, of whom 368 patients (60%) were treated with a DOAC and 241 patients (40%) with VKA, respectively. Thirty nine patients (6.4%) out of these received OAC for the first time without a diagnosis of atrial fibrillation or venous thromboembolism.

### Effectiveness and Safety Outcomes

Table 2 displays the number of effectiveness and safety outcome events occurring between 30 and 180 days after the procedure and the respective unadjusted event rates per 100 person-years according to postprocedural anticoagulation regime. Mechanical complications by artificial heart valve, ischemic stroke/SE events and MACE were generally rare, with slightly higher event rates in the treatment groups with mono or dual antiplatelet therapy. The highest event rates of death from all cause were observed in patients with no anticoagulation, followed by all treatment groups containing oral anticoagulation. After adjusting for baseline confounders (age, gender, renal insufficiency, history of PCI/Stenting, atrial fibrillation, history of any bleeding event, history of myocardial infarction <12 months, congestive heart failure, beta-blocker, dementia), in comparison to single antiplatelet therapy the risk of all-cause mortality was not significantly different in patients with DAPT (HR 0.75, 95% CI 0.50–1.14, *p* = 0.18). OAC containing regimes were associated with a significantly increased risk of all-cause mortality (HR 1.40, 95% CI 1.03–1.90, *p* = 0.03), as was no anticoagulation (HR 3.95, 95% CI 2.95–5.27, *p* < 0.0001).

**Table 2:** Event numbers and rates (per 100 person-years) of efficacy and safety endpoints according to post-TAVR anticoagulant treatment.

	**>30 days up to 6 months after procedure**											
	**Monotherapy**		**DAPT**		**OAC mono**		**OAC duo**		**OAC triple**		**No anticoagulation**	
	***N* events**	**Event rate**	***N* events**	**Event rate**	***N* events**	**Event rate**	***N* events**	**Event rate**	***N* events**	**Event rate**	***N* events**	**Event rate**
Ischemic stroke/SE	16	2.3	10	2.4	<5	n.a.	9	2.2	<5	n.a.	5	1.1
Mechanical complication by artificial heart valve	12	1.7	11	2.6	5	1.6	5	1.2	<5	n.a.	6	1.3
MACE	29	4.2	18	4.3	10	3.3	8	2	<5	n.a.	6	1.3
All-cause mortality	72	10.6	32	7.7	66	22.6	59	14.8	9	18	140	44.8
Combined bleeding endpoint (intracranial/extracranial/ gastrointestinal bleeding)	19	2.8	29	7	19	6.4	26	6.5	9	18.2	16	3.6
Intracranial bleeding	6	0.9	5	1.2	7	2.3	7	1.7	<5	n.a.	<5	n.a.
Extracranial bleeding	6	0.9	9	2.1	<5	n.a.	7	1.7	<5	n.a.	5	1.1
Gastrointestinal bleeding	9	1.3	15	3.6	10	3.3	13	3.2	<5	n.a.	11	2.4

*DAPT, dual antiplatelet therapy; MACE, major adverse cardiac event; OAC, oral anticoagulation; SE, systemic embolism*.

The bleeding events were mainly driven by gastrointestinal bleedings. Event rates were highest in patients with OAC triple and lowest in patients with single antiplatelet therapy. In patients with no anticoagulation, event rates for bleeding were generally low and comparable with those treated with single antiplatelet inhibition.

### Sensitivity Analysis

When using a time period of 120 days after the last preprocedural prescription to detect a postprocedural drug prescription for patients with OAC prior to TAVR and no OAC prescription within the 90 days interval, a total of 14 patients (4.0%) were ultimately issued a follow-up prescription for OAC and 339 patients (96.0%) still remained without prescription. For 196 patients who received single antiplatelet therapy before TAVR and no antithrombotic prescription within the 90 days postprocedural interval, 16 (8.2%) patients were prescribed aspirin or clopidogrel during the modified observation period.

## Discussion

The present study is the first to report anticoagulant treatment regimens after TAVR in a large real-world cohort. One third of the patients received an OAC containing regime with a DOAC portion of 64%, 29% of patients received antiplatelet monotherapy, 18% DAPT and 19% had no antiplatelet or anticoagulant drug prescription. Accordingly, almost two thirds of the patients were not treated according to current guideline recommendation during the study period between 2014 and 2018 with undertreatment or use of DOAC. Even from the perspective of the updated 2021 ESC/EACTS guidelines, almost 40% of patients continue to fall into this category. Furthermore, about 40% of patients with OAC prior to TAVR had no OAC prescription within the first 90 days after procedure. The risk of all-cause mortality was substantially increased in patients without any antiplatelet and anticoagulant therapy after TAVR.

So far data on clinical reality of anticoagulant treatment after TAVR are lacking. Several large TAVR registries assessed anticoagulant drugs (17–19). However, data have not been systematically reported and were only assessed at discharge from the interventional center which might substantially differ from the follow-up treatment in primary care (20).

In our real-world cohort, about 30% of patients had antiplatelet monotherapy. Although our outcome data, very recent trial evidence and guideline recommendations suggests that this might be preferable to DAPT due to a better safety profile (10, 21–24), it was in discrepancy to current guideline recommendations during our study period between 2014 and 2018 (9, 25). The majority of patients with postprocedural antiplatelet monotherapy did not have any anticoagulation prior to TAVR and had a clinical low risk profile for bleeding. This might suggest that the underlying reason for the former undertreatment with antiplatelet monotherapy was likely uncertainty due to the lack of trial evidence rather than concern for bleeding risk, and underlines the importance of controlled trials to improve adherence to recommendations.

Clear recommendations on additional antiplatelet drugs are lacking for patients with an indication for OAC after TAVR. In our cohort about 7% of patients with OAC were treated with a triple therapy which is comparable to discharge treatments in TAVR registries (17, 19). Importantly, these patients had an almost 3-fold increased risk of major bleeding when compared to OAC mono or dual therapy regimes. Following the paradigm shift away from triple therapy in the post PCI setting (26–29), our findings support restraint recommendations on the use of triple therapy also in patients after TAVR. This is of relevance since only a minority of our patients with triple therapy had justifying conditions like acute coronary syndrome or PCI.

OAC usually is a chronic therapy, particularly in the majority of patients with atrial fibrillation, and termination is accompanied by an increase in embolic risk (30). Almost 40% of patients with OAC prior to TAVR had no OAC prescription after TAVR, with higher rates in VKA than in DOAC treated patients. This termination rate is more than 2-fold higher when compared to data reported for patients with atrial fibrillation undergoing PCI (31). A potential explanation is more severe access site complications in TAVR compared to PCI, albeit we do not have data available on this. Nonetheless, access site complication should not preclude interruption of OAC for up to 90 days and further study is warranted to elucidate underlying causes of OAC termination after TAVR.

There is only little evidence regarding the use of DOAC in TAVR patients. Importantly, arecent meta-analysis reported a more protective effect of VKA compared to DOAC in post-TAVR patients on disabling or non-disabling stroke (32). The majority of our patients (64%) with OAC after TAVR used DOACs, both patients with *de-novo* OAC prescription and patients with OAC prior to TAVR, which is even higher than reported in TAVR registries (17, 33). Accordingly, clinical practice seems to move toward use of DOAC after TAVR, which is also supported by current trial evidence. Recently, results of the ATLANTIS trial have shown that in patients with an indication for oral anticoagulation, the use of apixaban after TAVR compared favorably with VKA on all endpoints (34). Moreover, in the ENVISAGE-TAVI AF trial, edoxaban was non-inferior to VKA for the primary composite endpoint of adverse clinical events, including all-cause death, myocardial infarction, ischemic stroke, systemic thromboembolism, valve thrombosis, and major bleeding according to the International Society on Thrombosis and Haemostasis (ISTH) definition (35).

A total of 926 patients (19.2%) were not prescribed any antiplatelet or anticoagulant therapy after TAVR which is remarkably higher than in discharge data of a current TAVR registry (19). When excluding patients with early death, still 15% of the total population had no postprocedural anticoagulant drug prescription. These patients were characterized by a higher Charlson Comorbidity Index, a higher HAS-BLED score, a higher proportion of chronic renal failure, and higher rates of bleeding events in the past compared with other regimes. Of note, 63% had prior antiplatelet or OAC therapy which means that the previous anticoagulatory therapy was actively terminated. The aforementioned comorbidity factors and renal insufficiency in particular, are associated with worse outcome after TAVR mainly due to periprocedural vascular and bleeding complications (36–39). This might be one potential explanation for termination of anticoagulatory medication. To what extent the termination of anticoagulatory therapy itself contributed to the increased risk of mid-term mortality cannot be concluded from our data. However, even after accounting for differences in comorbidity, patients with no postprocedural anticoagulatory treatment had the highest mortality across groups. Since postprocedural MACE in these patients were not higher than in the other treatment groups, an overall increased fragility must be assumed in this patient clientele contributing to cardiovascular and non-cardiovascular mortality risk. Since low-dose aspirin is freely available in pharmacies in Germany, it could be argued that over-the-counter use of this medication has led in part to an overestimation of individuals with no anticoagulation. However, 18.4% of patients with no anticoagulation were prescribed low-dose aspirin by their general practitioner before TAVR. Since reimbursement of aspirin is possible in Germany for justified indications, it is highly unlikely that a relevant proportion of these patients do not seek a prescription and start paying for low-dose aspirin themselves as an over-the-counter drug after TAVR. Furthermore, analyses from the German Drug Prescription Report demonstrated that the overall prescription of antiplatelet agents has remained constant in recent years, indicating an existing continuity in the follow-up prescription of antiplatelet drugs such as aspirin (40, 41). More importantly, it has already been shown for other European countries where low-dose aspirin is available without prescription that the level of potential misclassification of low-dose aspirin exposure due to unrecorded over-the-counter use appears to be low in Healthcare databases (42).

### Strength and Limitations

The strength of this study is the large and representative sample size reflecting almost 10% of the German statutory health insured population, and the data completeness with respect to follow-up and drug prescriptions. However, some limitations are inherent to the particular type of data source. Accuracy of patient characteristics depends on quality of coding. Since our conclusions are not dependent on exact absolute frequencies of comorbidities and coding errors may be similar across exposure groups, moderate inaccuracies in coding will not meaningfully influence conclusions. Clinical details of the postprocedural in-hospital course after TAVR can impact decisions on anticoagulatory treatment and might not be accurately reflected in coded diagnosis, for instance regarding access site status. Hence, our findings are mainly descriptive rather than exploratory. Furthermore, the exact start of the postprocedural anticoagulatory treatment regime cannot be exactly assessed with the available data and might differ from the prescription date. Hence, we pragmatically excluded outcome events within the first 30 days after TAVR (Supplementary Table 2) because the temporal association to the treatment regime is unclear. By using prescription claims data for defining treatment regimens it is not possible to detect termination of drugs before 90 days since most prescriptions provide drug supply for 90 days. Additionally, a switch of individual drugsor regimes within this early time can also not be accurately detected. Overall, this will lead to “undefined regimes” or an overestimation of total anticoagulatory drug intake. In consequence, the substantial undertreatment with respect to guideline recommendations observed in our cohort would be even more pronounced. In contrast, there was no evidence for relevant underestimation of treatments in our sensitivity analysis. Lastly, we cannot totally exclude over-the-counter use of low-dose aspirin in some cases, as it is freely available in pharmacies in Germany.

## Conclusions

This large real-world data analysis demonstrates deviations from guideline recommendations on anticoagulatory treatment after TAVR in more than two thirds of patients. Recent and ongoing randomized trials (AVATAR [NCT02735902], AUREA [NCT01642134]) will provide evidence on open questions such as DOAC use and necessity of a second antiplatelet drug which might contribute to improved guideline adherence in the future. However, the high rate of patients without any anticoagulant therapy after TAVR including 36% with prior OAC is of major clinical concern and needs further study since patients do have a considerable mortality risk.

## Data Availability Statement

The data used in this study cannot be made available in the article, the supplemental files, or in a public repository due to German data protection laws (Bundesdatenschutzgesetz). To facilitate the replication of results, anonymized data used for this study are stored on a secure drive at the InGef - Institute for Applied Health Research Berlin. Access to the raw data used in this study can only be provided to external parties under the conditions of a cooperation contract and can be accessed upon request, after written approval ( info@ingef.de), if required.

## Ethics Statement

Ethical review and approval was not required for the study on human participants in accordance with the local legislation and institutional requirements. Written informed consent for participation was not required for this study in accordance with the national legislation and the institutional requirements.

## Author Contributions

CH and RP: conceptualization. CH, ML, JW, HW, SB, and RP: methodology and investigation. CH: writing—original draft preparation and visualization. RP: writing—review and editing. All authors have read and agreed to the published version of the manuscript.

## Conflict of Interest

ML and JW were employed by company InGef-Institute for Applied Health Research Berlin GmbH. The remaining authors declare that the research was conducted in the absence of any commercial or financial relationships that could be construed as a potential conflict of interest.

## Publisher's Note

All claims expressed in this article are solely those of the authors and do not necessarily represent those of their affiliated organizations, or those of the publisher, the editors and the reviewers. Any product that may be evaluated in this article, or claim that may be made by its manufacturer, is not guaranteed or endorsed by the publisher.
