# Supplementary material for: Real-World Anticoagulatory Treatment After Transcatheter Aortic Valve Replacement: A Retrospective, Observational Study on 4,800 Patients
Source: Front Cardiovasc Med. 2021 Dec 23;8:780762. doi: 10.3389/fcvm.2021.780762 (PMC8733398; doi:10.3389/fcvm.2021.780762)
Supplement: Supplementary file 1 [file Table_1.DOCX]

| **Endpoint** | **ICD-10 GM code** |
| --- | --- |
| Ischemic stroke | I63* |
| Systemic embolism | I74* |
| Mechanical complication by artificial heart valve | T82.0 |
| MACE  Myocardial infarction  Ischemic stroke | I21*/I22*  I63* |
| *Intracerebral bleeding*  Subarachnoid haemorrhage  Intracerebral bleeding  Other non-traumatic intracranial haemorrhage  Focal brain injury  Epidural haemorrhage  Traumatic subdural haemorrhage  Traumatic subarachnoidal haemorrhage | I60*  I61*  I62*  S06.3  S06.4  S06.5  S06.6 |
| *Extracranial major bleeding*  Acute posthaemorrhagic anaemia  Haemothorax  Conjunctival haemorrhage  Retinal haemorrhage  Vitreal haemorrhage  Recurrent and persistent haematuria  Haemorrhage from respiratory passages  Unspecified haematuria  Haemorrhage, not elsewhere classified  Haemarthrosis  Other abnormal uterine and vaginal bleeding | D62*  J94.2  H11.3  H35.6  H43.1/H45.0  N02  R04  R31  R58  M25.0  N93 |
| *Gastrointestinal bleeding*  Gastric ulcer bleeding  Duodenal ulcer bleeding  Peptic ulcer bleeding  Jejunal ulcer bleeding  Gastritis with bleeding  Oesophageal varices with bleeding  Haemorrhage of anus and rectum  Hematemesis | K25.-0,2,4,6  K26.-0,2,4,6  K27.-0,2,4,6  K28.-0,2,4,6  K29.-0  I85.0  K62.5  K92.-0,1,2 |
