# Supplementary material for: Real-World Anticoagulatory Treatment After Transcatheter Aortic Valve Replacement: A Retrospective, Observational Study on 4,800 Patients
Source: Front Cardiovasc Med. 2021 Dec 23;8:780762. doi: 10.3389/fcvm.2021.780762 (PMC8733398; doi:10.3389/fcvm.2021.780762)
Supplement: Supplementary file 2 [file Table_2.pdf]

Supplementary Table 2: Event numbers and rates (per 100 person-years) of efficacy and safety endpoints < 30 days after procedure according to post-TAVR anticoagulant treatment.

	**< 30 days after procedure**											
	**Monotherapy**		**DAPT**		**OAC mono**		**OAC duo**		**OAC triple**		**No anticoagulation**	
	N events	Event rate	N events	Event rate	N events	Event rate	N events	Event rate	N events	Event rate	N events	Event rate
**Ischemic stroke/SE**	7	1.0	< 5	n.a.	5	1.6	8	2.0	< 5	n.a.	10	2.2
**Mechanical complication by artificial heart valve**	< 5	n.a.	< 5	n.a.	< 5	n.a.	< 5	n.a.	< 5	n.a.	< 5	n.a.
**MACE**	9	1.3	< 5	n.a.	6	2.0	9	2.2	< 5	n.a.	11	2.5
**Combined bleeding endpoint (intracranial/extracranial/ gastrointestinal bleeding)**	12	1.8	< 5	n.a.	6	2.0	12	3.0	< 5	n.a.	9	2.0

DAPT, dual antiplatelet therapy; MACE, major adverse cardiac event; OAC, oral anticoagulation; SE, systemic embolism
